# Another human case of equine morbillivirus disease in Australia.

**DOI:** 10.3201/eid0201.960112

**Published:** 1996


### Another Human Case of Equine Morbillivirus Disease in Australia

Another human case of equine morbillivirus (EMV) disease has occurred in Australia. The patient was a 35-year-old farmer, who lived near Mackay, in northern Queensland. He died in the Royal Brisbane Hospital on October 21, 1995 (1). The patient was probably infected with the novel virus 12 months before his death—approximately the time of the first reported outbreak of EMV.

A hitherto unknown infectious disease, EMV was first observed in Brisbane, Queensland, in September 1994, when an outbreak of acute respiratory disease in horses at three stables in Hendra, a Brisbane suburb, was reported (2). In a 2-week period, 14 racehorses and two persons at the stable contracted the disease. One of the human cases and some of the equine cases were fatal. A total of 21 horses were infected with the virus. Fourteen horses died as a result of clinical illness (they either died from the infection or were euthanized). The remaining horses had either symptomatic or asymptomatic infection and were euthanized.

The cause of infection in the recent case has been confirmed as EMV at the Australian Animal Health Laboratory in Geelong through the testing of samples taken from the patient before he died (I. Douglas, Australian Communicable Disease Service; PROMED).

The Mackay patient was married to a veterinary surgeon. The couple bred horses and grew sugar cane. In August 1994 (a month before the outbreak of EMV in southern Queensland), two horses died on the couple's property. The veterinary surgeon, assisted by her husband, performed autopsies on the two animals. The diagnoses, based on these autopsies, were "avocado poisoning" and "brown snake bite," respectively (1).

In August-September 1994, soon after the death of the horses, the husband became ill with a mild meningoencephalitis, which improved with antibiotics. Cerebrospinal fluid examination showed a neutrophilic pleocytosis suggestive of a viral infection (1). Serum collected at the time of the examination and stored was found to contain a low but significant titer of antibody to EMV.

The patient appeared to have recovered; however, he was admitted to the hospital 5 weeks before his death with signs of encephalitis. Evidence of EMV infection included a high serum neutralizing antibody titer against the virus and a positive polymerase chain reaction (PCR) test of cerebrospinal fluid collected before his death. Tests of autopsy specimens confirmed the infection.

Direct fluorescence antibody and PCR tests of fixed tissue blocks from one of the horses at the Australian Animal Health Laboratory have confirmed that it was infected with EMV (I. Douglas, PROMED). However, it is likely that both horses were infected.

The Mackay patient's symptoms were predominantly neurologic. He displayed no respiratory symptoms until aspiration pneumonia developed. By contrast, the major clinical symptoms of EMV in Hendra were respiratory.

No recent outbreaks of clinical illness have been reported in the horses on the Mackay property—or elsewhere in Queensland—since the 1994 outbreak. Also, investigation has not shown a link between the horses on the Mackay property and those in the Hendra stables. A serologic survey of over 2,000 horses, undertaken in 1994 after the Hendra outbreak, yielded negative results. That survey included more than 200 horses in the Mackay/Rockhampton/Townsville areas. Similarly, the Queensland veterinary authorities have obtained samples from more than 3,000 animals from 294 populations (including farms, race meetings, and horse events) since October 23, 1995; 2,349 of the samples have been tested, all with negative results. Moreover, blood samples recently taken from all the domestic animals on the Mackay property, including approximately 90 horses, have been tested for virus by the Animal Health Bureau, Queensland Department of Primary Industry and Energy. All results were negative.

An extensive epidemiologic investigation is being conducted by the Queensland Department of Health and the Department of Primary Industry and Energy. All persons who may have had exposure to the virus in either episode have been tested for EMV infection and had negative results (1). No serologic evidence of further human infection has been found.

No human-to-human transmission of EMV has been reported. It is believed that the disease is spread through contact with the body fluids of infected sick or dying animals.

**References**

1. Allworth T, O'Sullivan J, Selvey L, Sheridan J. Equine morbillivirus in Queensland. Communicable Diseases Intelligence 1995;19:575.
2. Murray K, Rogers R, Selvey L, Selleck P, Hyatt A, Gould A, et al. A novel morbillivirus pneumonia of horses and its transmission to humans. Emerging Infectious Diseases 1995;1:31-3.

**Sandra Lane**
Case Western Reserve University
Cleveland, Ohio, USA
E-mail: sxl45@po.cwru.edu

### Social Science and the Study of Emerging Infectious Diseases

Topics related to emerging and reemerging infectious diseases attracted a considerable audience at the annual meeting of the American Anthropological Association, November 15–19, 1995, in Washington, D.C. The meeting had a separate session entitled "Emerging and Reemerging Infectious Diseases: Biocultural and Sociocultural Approaches."

The session brought together anthropologists interested in and working on emerging infectious diseases from various subdisciplinary perspectives. Presentations were made on the following subjects: outline of a research agenda, deforestation and the emergence of infectious diseases in the rain forests of Papua-New Guinea, the cholera epidemic in Latin America, evolutionary aspects of emergent infections, societal impacts of the test for acquired immunodeficiency syndrome, compliance and iatrogenesis in tuberculosis treatment in the United States, patchwork policies that affect long-term treatment of tuberculosis in Nepal and Uganda, the reemergence of schistosomiasis in Egypt, dengue control in Latin America, cultural and political ecologic models of emergent infections, and the politics of leprosy eradication. Abstracts are available from the conference organizers, listed below.

Anthropologists interested in international health and the social science aspects of infectious diseases are organized in a working group called the International Health and Infectious Disease Study Group of the Society of Medical Anthropology (American Anthropological Association). Requests to subscribe to this group's newsletter can be sent to

**Johannes Sommerfeld**
Harvard Institute for International Development
Cambridge, Massachusetts, USA
Phone: 617-495-9791
E-mail: jsommerf@hiid.harvard.edu

### WHO Establishes New Rapid-Response Unit for Emerging Infectious Diseases

The World Health Organization (WHO) has established a new rapid-response unit to control and prevent the growing incidence of new and reemerging diseases worldwide. The unit's focus will be improved containment of disease outbreaks, such as that caused by the deadly Ebola virus, which struck Zaire in 1995.

The WHO unit will be called the Division of Emerging Viral and Bacterial Diseases Surveillance and Control (EMC). It will be capable of mobilizing staff from WHO headquarters in Geneva and from the organization's regional offices.

In addition to mobilizing WHO's own technical staff and expertise, EMC will coordinate the activities of the agency's traditional partners, for example, its international network of collaborating centers, bilateral donors, expert advisers, and nongovernmental organizations.

Teams equipped to implement epidemic control measures will be placed on-site within 24 hours' notification of an outbreak. This strategy, when implemented in Zaire, not only rapidly contained the recent Ebola outbreak but also prevented its spread to Kinshasa, the capital city of 2 million.

Among EMC's goals are 1) to strengthen local surveillance and disease control so that countries can develop the early warning systems needed to detect emerging or reemerging diseases through innovative field epidemiology and public health laboratory training programs and 2) to continue WHO's activities in developing a network of public health laboratories to strengthen regional and international collaboration in outbreak detection and control.

EMC will continue to expand WHO's network—termed WHONET—that detects and monitors antibiotic resistance worldwide. WHO will use the information collected to continue to advocate research and development of new antibiotics to replace those that are no longer effective.

